# Negative Pressure Wound Therapy (NPWT): Our Experience in Pakistan With Locally Made Dressing

**DOI:** 10.7759/cureus.9464

**Published:** 2020-07-29

**Authors:** Haroon Ur Rashid, Mamoon Rashid, Saad Ur Rehman Sarwar, Ibrahim Khan, Nasir Khan, Noshi Bibi

**Affiliations:** 1 Plastic Surgery, Shifa International Hospital, Islamabad, PAK; 2 Plastic and Reconstructive Surgery, Shifa International Hospital, Islamabad, PAK

**Keywords:** negative pressure wound therapy, vacuum-assisted closure, wound management, complex compound fractures, atmospheric pressure device, kci concepts inc

## Abstract

Introduction

Worldwide numbers of patients suffering from complex wounds appear to increase annually. These patients present with acute, sub-acute and chronic wounds which can be difficult to manage. Management of these patients typically requires a multi-disciplinary approach by a plastic surgeon, orthopaedic surgeon and infectious disease control team. Despite the advent of numerous new techniques and technologies, negative pressure wound therapy (NPWT) remains a cornerstone to the management of complex wounds. We present our experience with NPWT in this study.

Methods

This is a retrospective study of 380 patients who were treated with NPWT in the last 10 years at a single center. We receive hundreds of infected wounds of limbs each year which are either post-traumatic or post-debridement. Frequency of dressing change, C-reactive protein levels, bacterial cultures, complication rate and cost of apparatus in each case were noted. All patients received systemic antibiotics during the treatment.

Results

We reviewed hospital data of 520 patients in which debridement was performed. Of the 520 patients derided, 380 patients were treated with NPWT, and included in study. Number of NPWT sessions was decided on the basis of wound status (adequate healthy granulation tissue, clinically improved circulation). A single session of NPWT dressing was applied in 84% (n = 320) patients, 8% (n = 31) patients needed two sessions of NPWT dressing, 6% (n = 24) patients had three sessions of NPWT dressing and only 1% (n = 5) patients did not respond to NPWT dressing. 78% (n = 297) patients had reduced levels of C-reactive protein levels and wound cultures were negative in 54% (n = 208) patients after application of NPWT dressing. Minor complications occurred in 0.7% (n = 3) patients due to occult osteomyelitis. In 0.5% (n = 2) patients, NPWT dressing was discontinued due to persistent leakage near a natural orifice. Sinus formation was seen in 6% (n = 23) patients who were treated with curettage and conventional dressings. The mean pain score on the verbal analogue scale was 3 out of 10. The mean cost of an NPWT dressing apparatus was 90 dollars.

Conclusion

We conclude from this study that NPWT dressing can be easily applied to any region of the body and it can be customized to the needs of patients from different socio-economic status.

## Introduction

Negative pressure wound therapy (NPWT) remains a useful adjunct in wound temporization. NPWT has many names: sub-atmospheric pressure device (SPD), topical negative pressure (TNP), vacuum-assisted closure (VAC), vacuum sealing technique (VST) and sealed surface wound suction (SSS) [1]. It was first described by Fleischmann et al. in 1993 [2]. Morykwas and Argenta were the first to investigate efficacy of NPWT in 1997 [3,4]. Since their original description, numerous studies have reinforced the importance of NPWT in the management of wounds and more recently as a bolster for flaps and grafts. In 1996, the first commercially available NPWT system was introduced by Kinetic Concepts, Inc./KCI USA, Inc., San Antonio, TX. The basic mechanism of NPWT is mechanotransduction and micro deformation leading to cellular proliferation under hypoxic stress providing an environment for angiogenesis [5]. NPWT has evolved and been modified in the last three decades to make it more cost effective and improve its portability [6,7]. The basic goals of an NPWT dressing are to accelerate wound healing by reducing edema, contraction at wound edges, granulation from the base of the wound and typically reduced dressing change frequency and hospital stay. We studied the results and efficacy of our own local made negative pressure dressing over the last 10 years. This model was introduced by S.A. Malik in the early nineties (unpublished data) in Pakistan.

## Materials and methods

This retrospective study was done in Shifa International Hospital Islamabad from January 2010 till January 2020. After approval from the ethical committee, a total of 380 patients included in the study. Patients had wounds of different etiologies and anatomical regions. Hospital operation room records and data of the last 10 years were gathered and all the details of debridement and NPWT cases were collected. Outcomes of NPWT dressings were noted in terms of the number of sessions required, C-reactive protein levels before and after therapy, bacterial cultures, complications and dressing cost in each case. All patients received systemic antibiotics according to tissue cultures. The inclusion and exclusion criteria were outlined as follows:

Inclusion criteria:

1. All dehisced wounds (Abdominoplasty, laparotomy, Post CABG sternal wounds)

2. Entero-cutaneous fistulas / hidradenitis wounds

3. Large open fractures lower limb / upper limb

4. Grafted wounds / large wounds waiting for flap coverage

5. Pressure sores and lymphedema patients

Exclusion criteria:

1. Patients in which follow-up was lost.

2. NPWT dressings with inadequate patients compliance

Apparatus details:

1. Portable phlegm suction machine (cost 55-60 dollars)

2. Yankauer suction set (cost 3-5 dollars)

3. Autoclaved sponge foam 1 cm thickness (150° C, 20 PSI, 30 minutes)

4. Ioban / Opsite transparent dressings (cost 8-9 dollars)

5. Two way / Y-shaped plastic cannulas for multiple sites drainage (cost 0.5-1 dollars)

Application sequence

After adequate debridement and meticulous hemostasis, the patient's surrounding skin was meticulously prepared. Hairs are removed first, Tincture of benzoin is applied to native skin to improve the sticking of dressing and as sealant. Autoclaved foam is cut according to the defect shape and size and placed in the wound. For sternotomy wounds, where pericardium is exposed a soft paraffin gauze layer is placed before applying foam over the wound. The Yankauer suction tube is fenestrated with scissors and placed over the foam. The tube is further covered with a piece of foam. The transparent sticking applied over it covering the native skin for at least 2 cm avoiding circumferential dressing. Mesentery of dressing is made along with the tube and skin to keep it airtight. Initially, low suction pressure is applied to see for the contraction of sponge foam and if any leak is there. For ischaemic wounds, grafted areas and over pericardium the suction pressure is kept in control (5-10 K pa, -50 to -60 mm Hg). For all other wounds the pressure is raised (15 to 20 K pa, -100 to -120 mm Hg). The first few hours are critical to monitor any ooze / haematoma collection within the dressing and blockage of tube or leakage. The dressing is changed after 48 to 72 hours. Photographic records are maintained and culture swabs are taken at every dressing change. If the granulation tissue is evident and the wound is healthy enough then coverage with graft or flap is planned.

## Results

A single session of NPWT dressing was done in 84% (n = 320) patients, 8% (n = 31) patients needed two sessions of NPWT dressing, 6% (n = 24) patients had three sessions of NPWT dressing and only 1% (n = 5) patients did not respond to NPWT dressing. In 83% (n = 317) patients the surrounding skin of wounds showed a significant reduction in inflammation and edema on clinical examination. 78% (n = 297) patients had reduced levels of C-reactive protein levels after application of NPWT dressing and wound cultures were negative in 54% (n = 208) patients before the definitive procedure. In 73% (n = 280) patients, NPWT was used as wound temporizing and stabilizing tool, whereas in the remaining 26% (n = 100) patients, NPWT was applied as an adjunct to a definitive procedure like grafting, flap coverage and direct closure (over the suture line). In this study, NPWT therapy was abandoned in 0.7% (n = 3) patients, which were complicated with occult osteomyelitis and deteriorated the wound bed. In 0.5% (n = 2) patients, NPWT dressing was discontinued due to persistent leakage near natural orifice. Sinus formation was seen in 6% (n = 23) patients who were conservatively managed with curettage and antiseptic dressings. The mean pain score on a verbal analogue scale was 3 out of 10. The mean cost of NPWT dressing apparatus was 90 dollars. Negative pressure was applied in a continuous manner in all patients. Table 1 shows outcomes in our patients.

**Table 1:** Outcome of our patients with NPWT dressings. NPWT: Negative pressure wound therapy

Parameters	Results		
C-reactive protein level after NPWT	Reduced in 78% (n = 297)	Increased in 18% (n = 68)	No change in 4% (n = 15)
Bacterial cultures after NPWT	No growth in 54% (n = 208)	Persistent in 40% (n = 152)	Change of spectrum in 5% (n = 20)
Number of NPWT dressings	Single session in 84% (n = 320)	Two sessions in 8% (n = 31)	Three sessions in 6% (n = 24)
Cost of NPWT dressings in dollars	Mean cost $90	Maximum cost $120	Minimum cost $60
Pain score (out of 10)	Mean 3	Maximum score 5	Minimum score 1
Complications	<1%		

Following are some of our cases which were included in this study. NPWT dressings were applied either on wound or skin grafted region. These cases are commonest form of scenarios we face in our daily practice. Each case shows one of the different regions.

**Figure 1:**
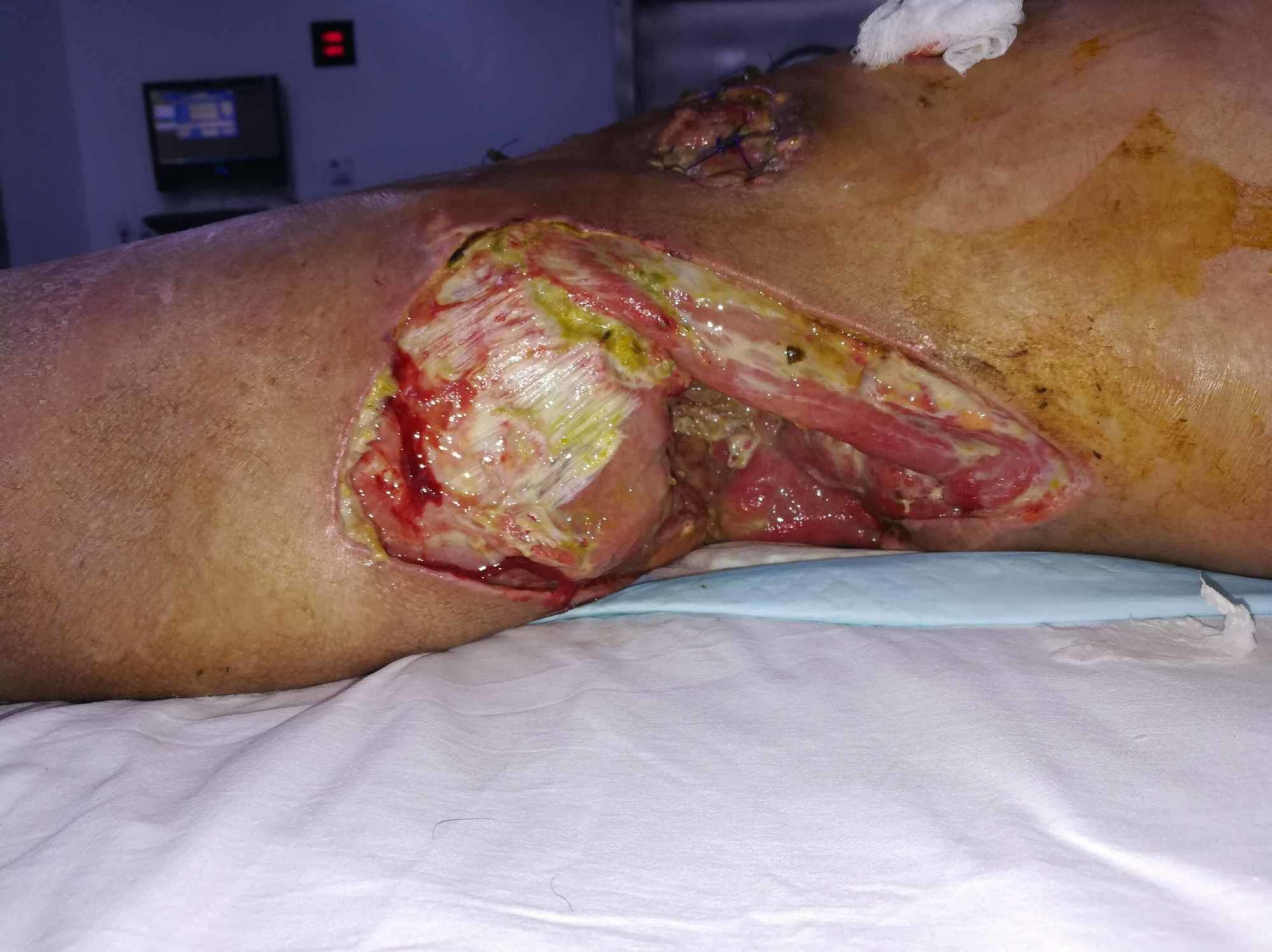
Case 1 is a 24-year-old young male presented with history of gun shots to abdomen and groin region, leading to necrotizing fasciitis.

**Figure 2:**
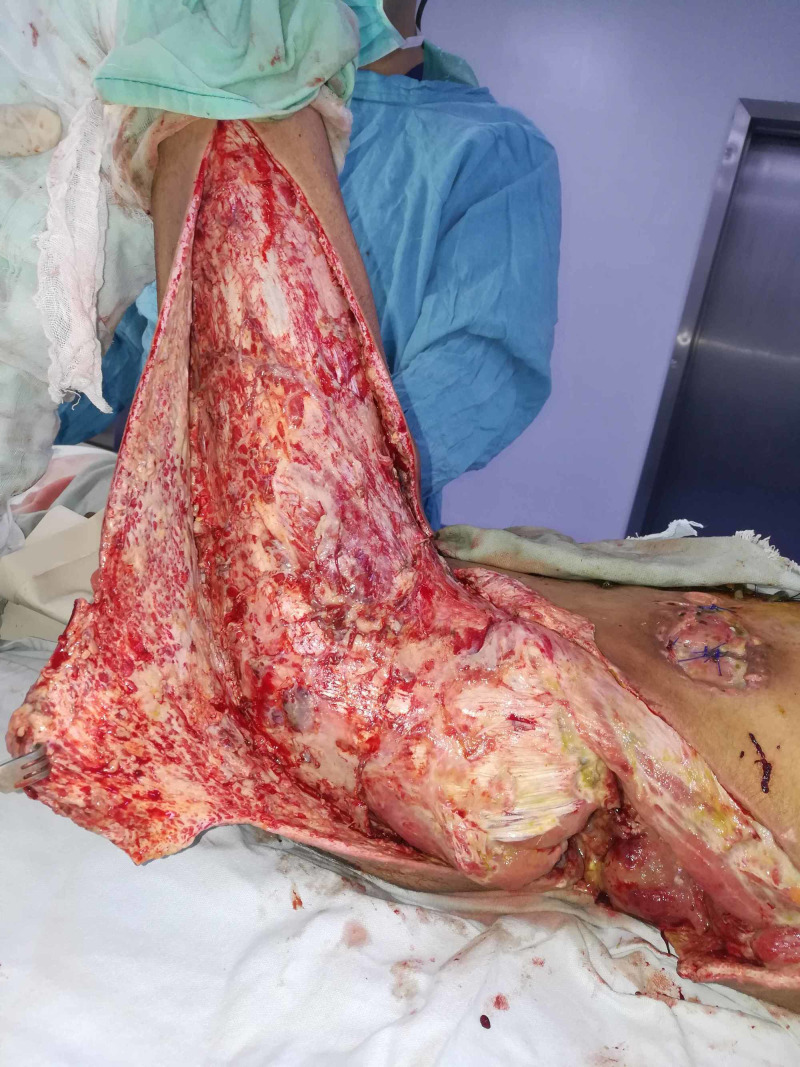
Case 1 picture showing extensive debridement done for removing the necrosed tissue.

**Figure 3:**
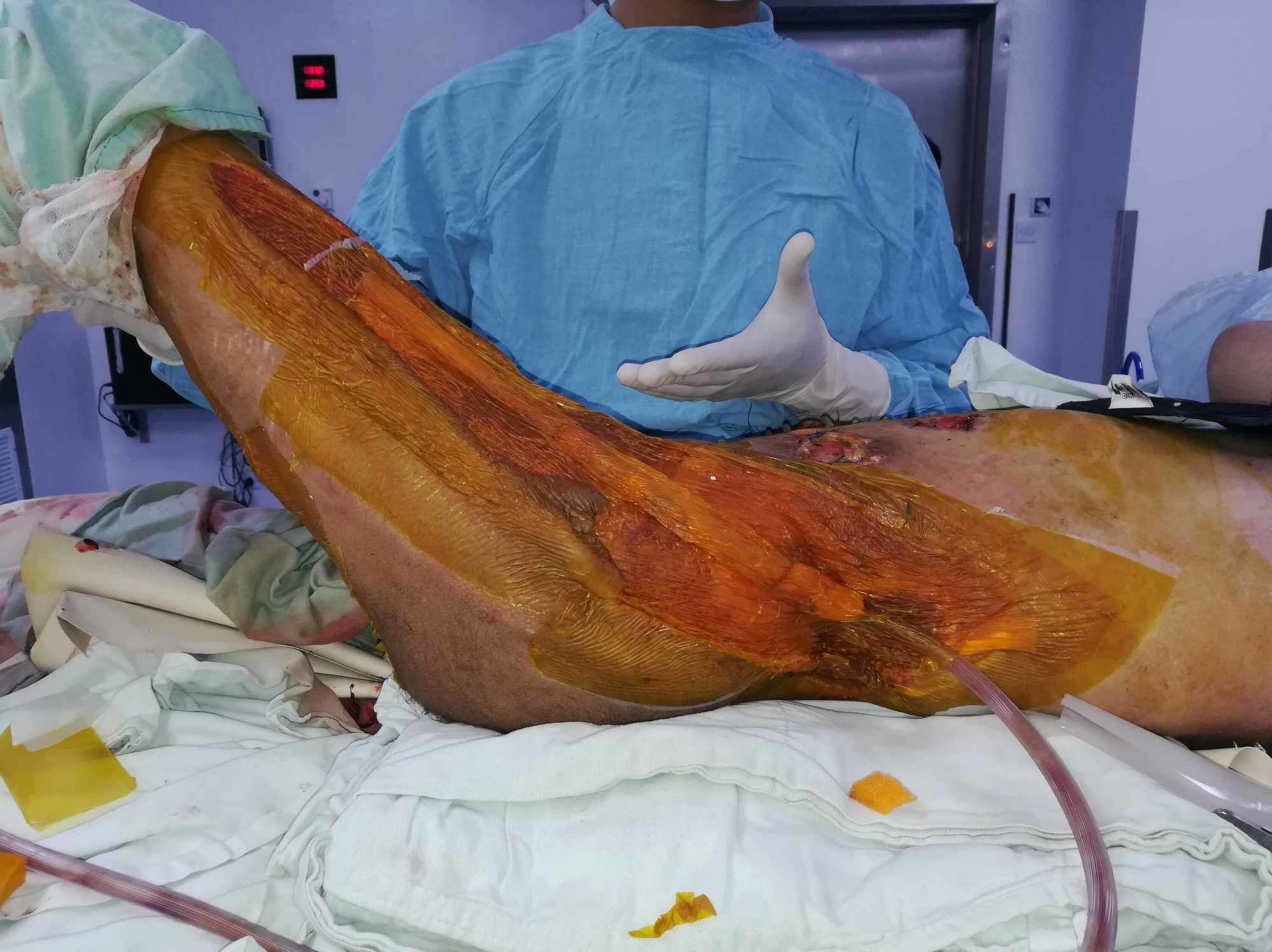
Case 1 picture showing a large NPWT dressing is applied to left thigh and groin region, dressing partially buried inside wound. NPWT: Negative pressure wound therapy

**Figure 4:**
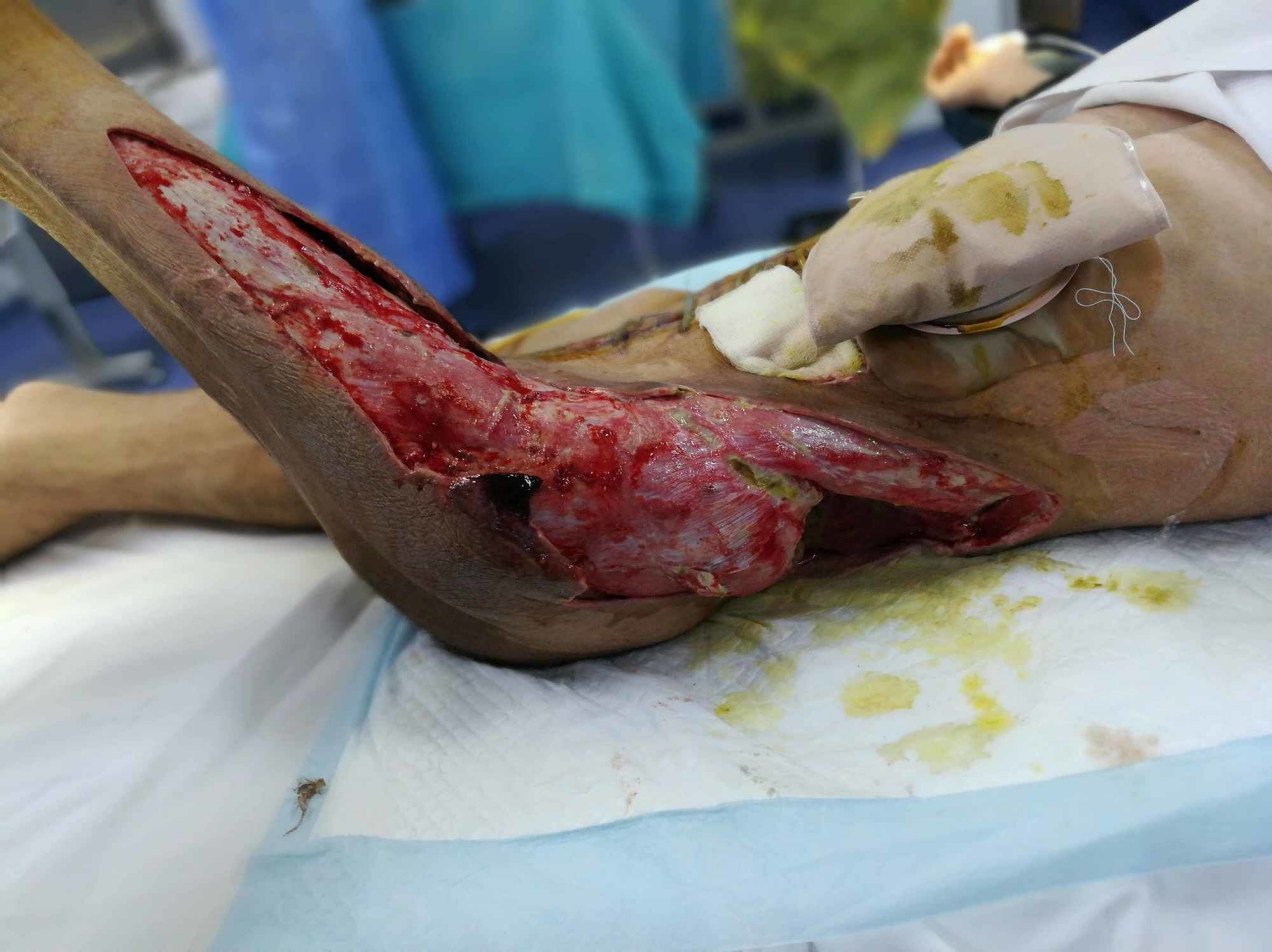
Case 1 picture showing after 48 hours of NPWT dressing, wound with reduced edema and improved local circulation. NPWT: Negative pressure wound therapy

Case 1 (Figures 1-4) is a young male presented to us with history of gunshot to abdomen and groin. He had multiple debridements done for necrotizing fasciitis and diversion colostomy done. His general health was optimized. NPWT was applied for 48 hours. After 48 hours, the wound bed has significant improvement in granulation tissue and reduced edema. Wound was covered with skin grafts.

**Figure 5:**
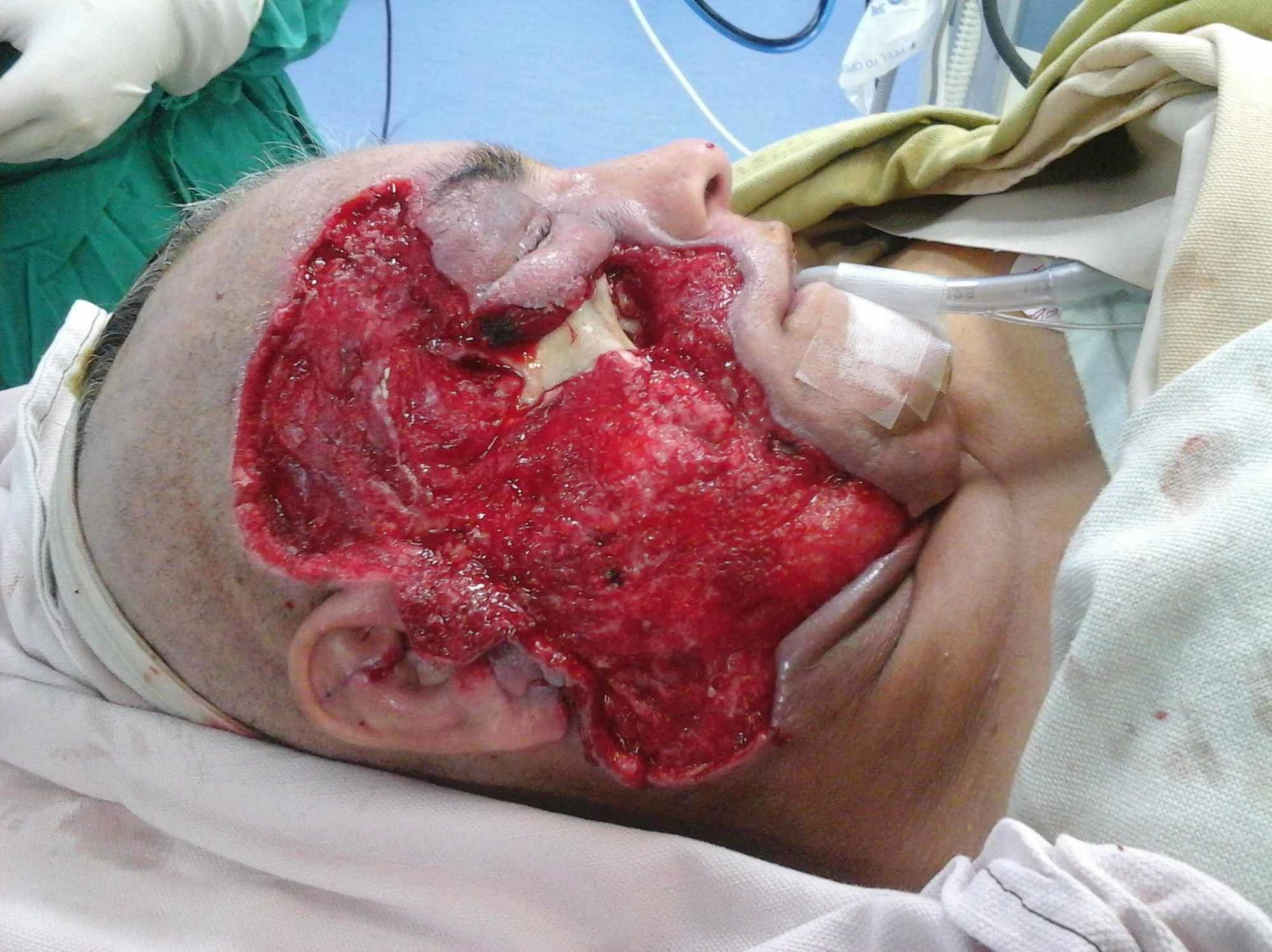
Case 2 picture showing a 40-year-old male presented with degloving injury to right side face with complete loss of soft tissue and exposed bone. The picture shows wound after multiple debridements.

**Figure 6:**
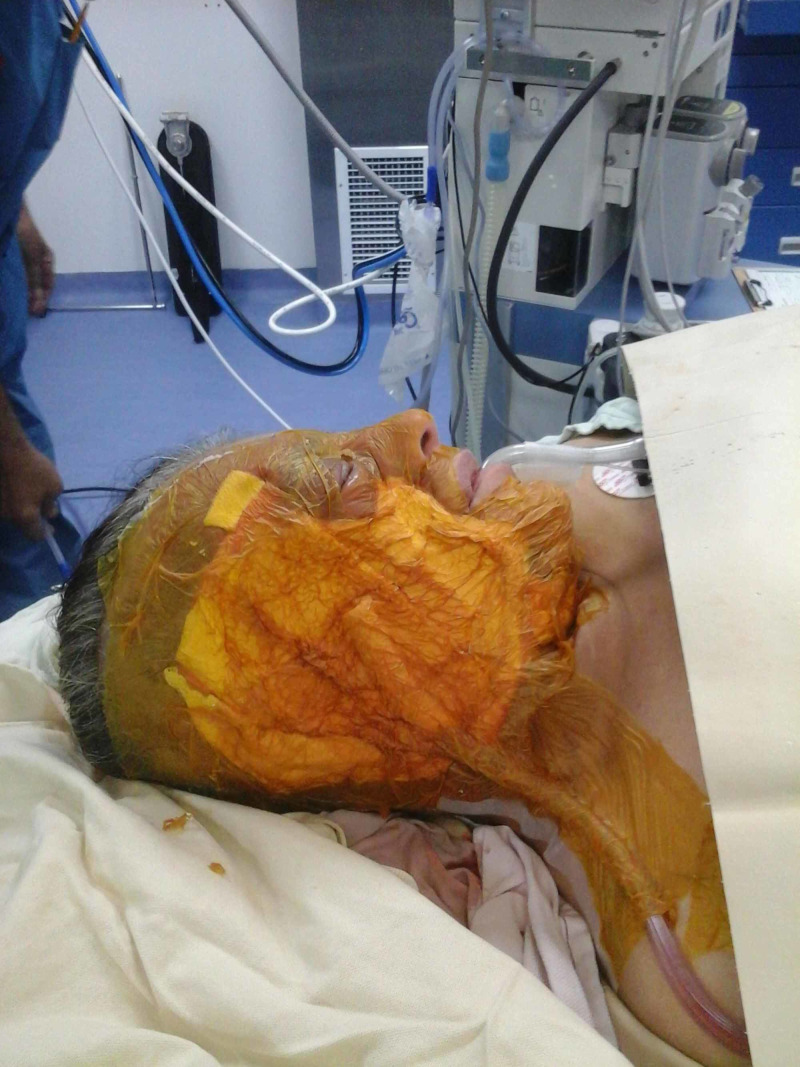
Case 2 picture showing a large NPWT dressing applied to the wound with orbital area spared. NPWT: Negative pressure wound therapy

**Figure 7:**
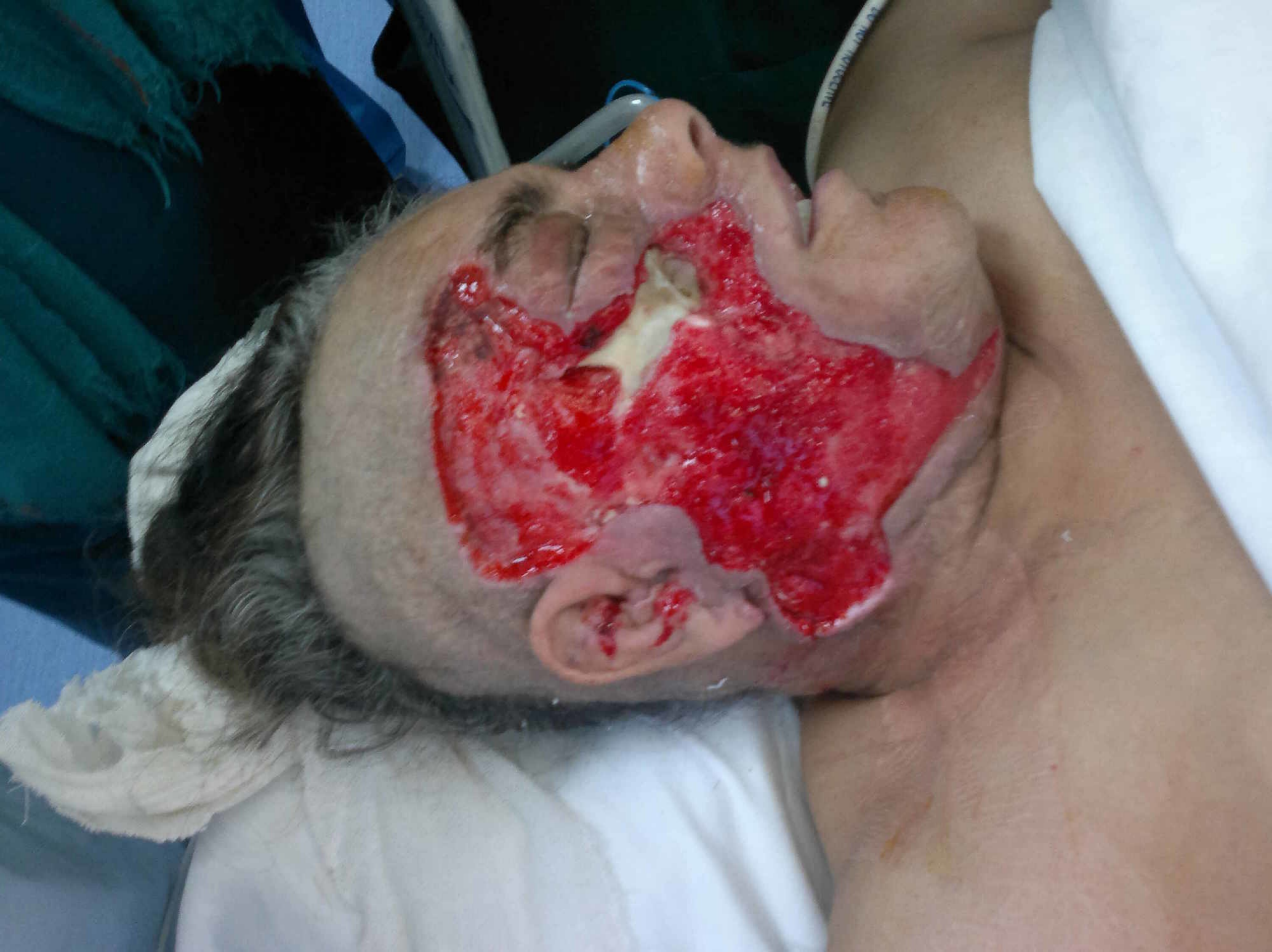
Case 2 picture showing after 48 hours of NPWT dressing, significant reduction in edema and improved granulation tissue at wound bed. Wound is ready for coverage. NPWT: Negative pressure wound therapy

**Figure 8:**
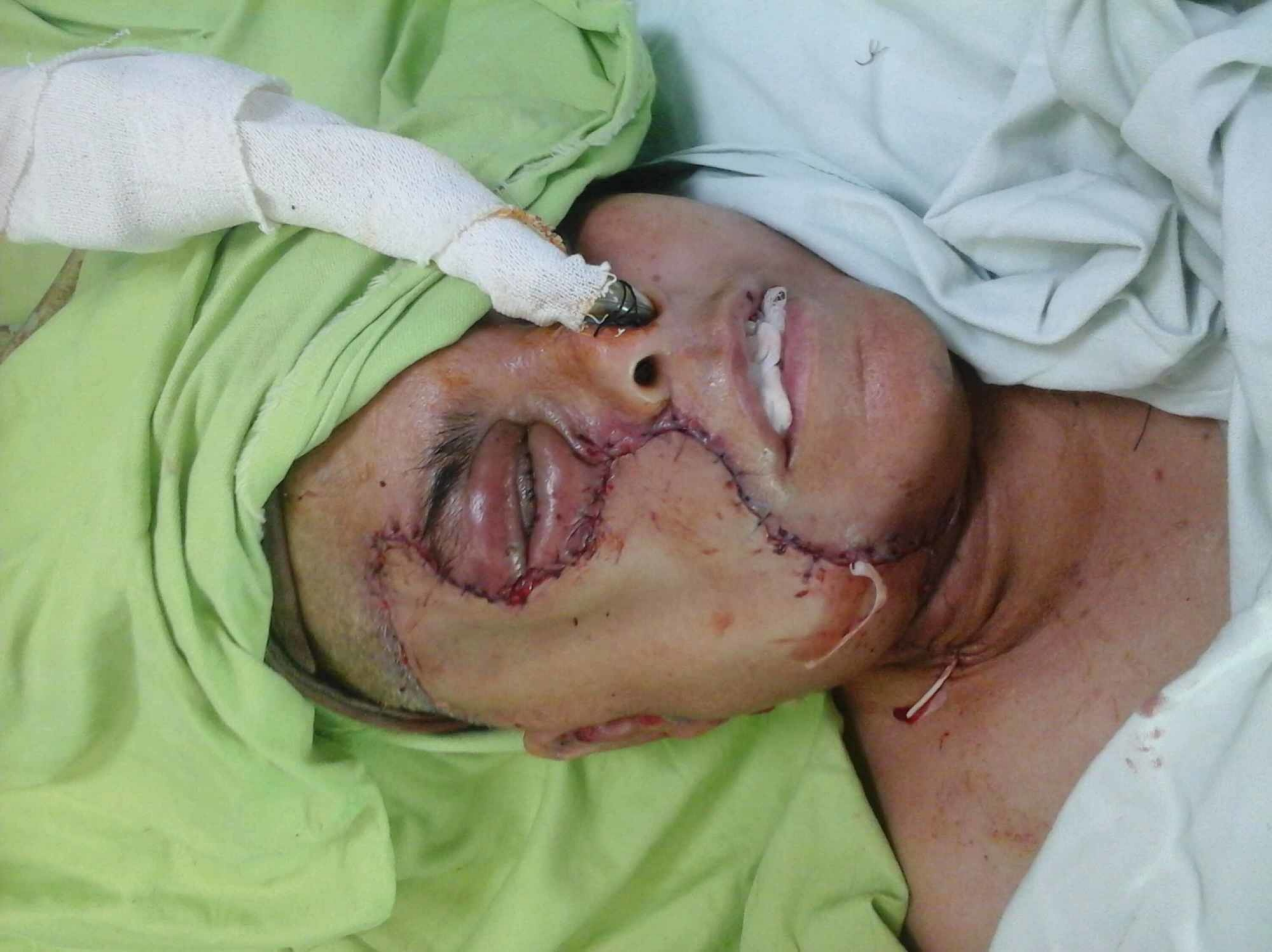
Case 2 picture showing a free anterolateral thigh flap used to cover the whole wound.

Case 2 (Figures 5-8) (Video 1) is a middle age male with history of degloving injury to right side face leading to complete loss of skin and soft tissue. After multiple debridements, NPWT is applied and there is significant improvement in local tissue vascularity and edema. A free antero-lateral thigh flap is used to cover the wound.

**Video 1:** Case 2 video showing NPWT dressing suction maintained. NPWT: Negative pressure wound therapy

**Figure 9:**
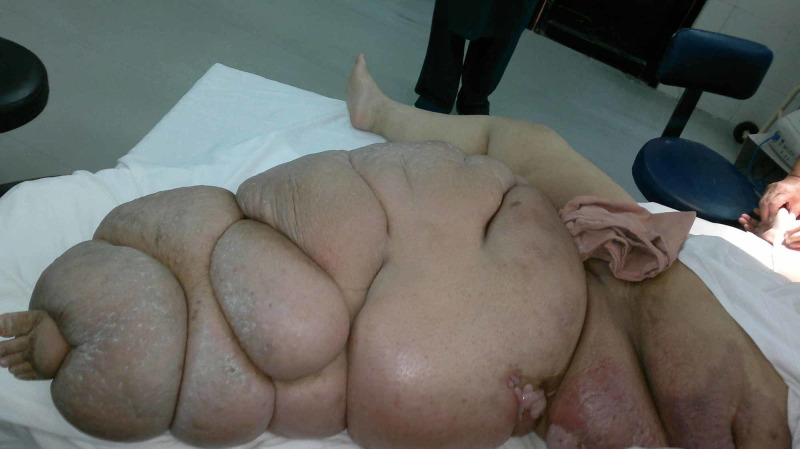
Case 3 picture showing a young female with elephantiasis left lower limb.

**Figure 10:**
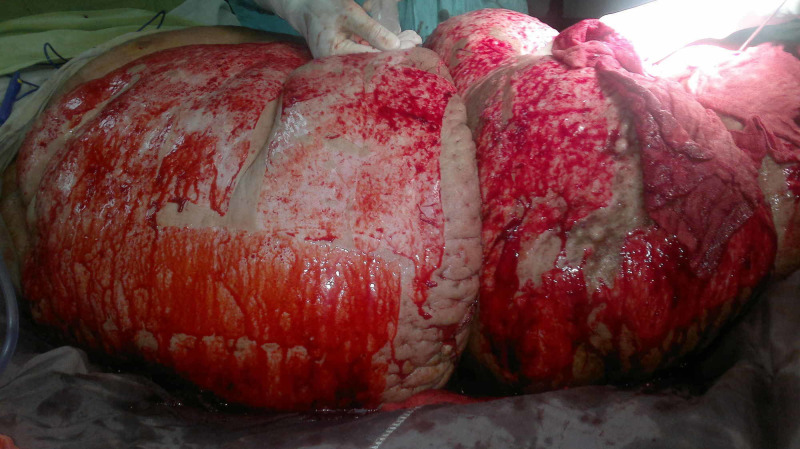
Case 3 picture showing skin graft taken at the start from same limb for later use.

**Figure 11:**
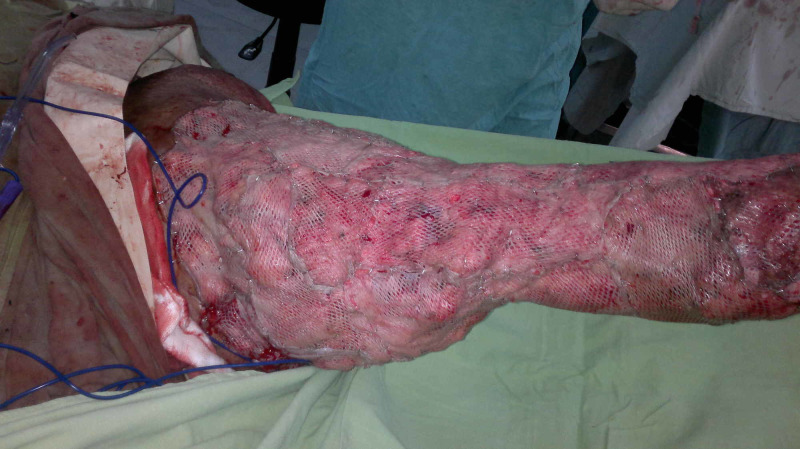
Case 3 picture showing Charles' procedure done. Skin graft taken at first stage is used to cover the whole limb.

**Figure 12:**
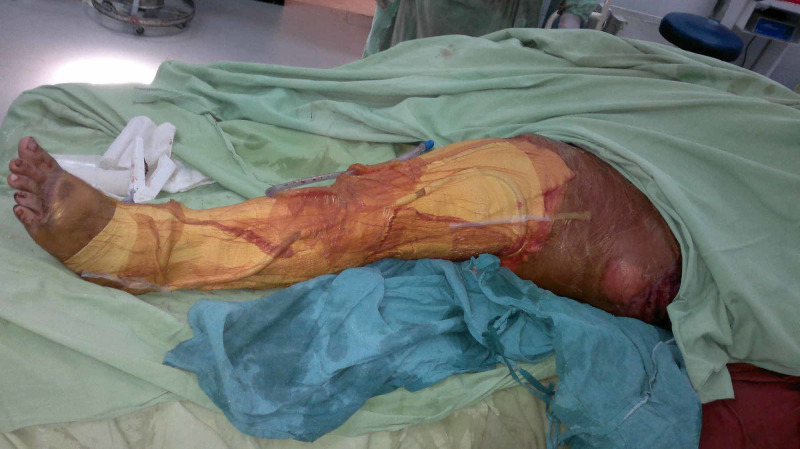
Case 3 picture showing well sealed large NPWT dressing over skin grafted wound. NPWT: Negative pressure wound therapy

**Figure 13:**
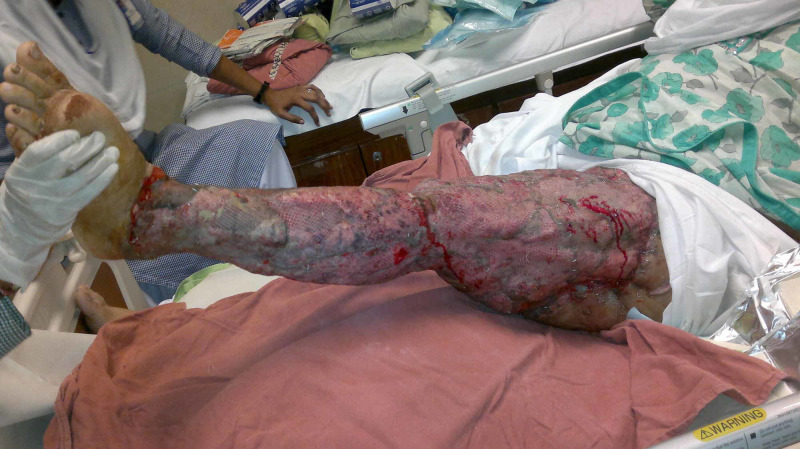
Case 3 picture showing NPWT dressing removed after 48 hours. Skin graft is well taken. NPWT: Negative pressure wound therapy

Case 3 (Figures 9-13) (Video 2) is a young female presented with elephantiasis left lower limb. She was booked for elective surgery (Charles' Procedure). Skin grafts are taken from the edematous leg and used for later coverage. A large NPWT dressing was applied over the grafted wound. She has very good recovery and well healed grafted skin.

**Video 2:** Case 3 video showing large NPWT dressing applied to skin grafted wound. NPWT: Negative pressure wound therapy

**Figure 14:**
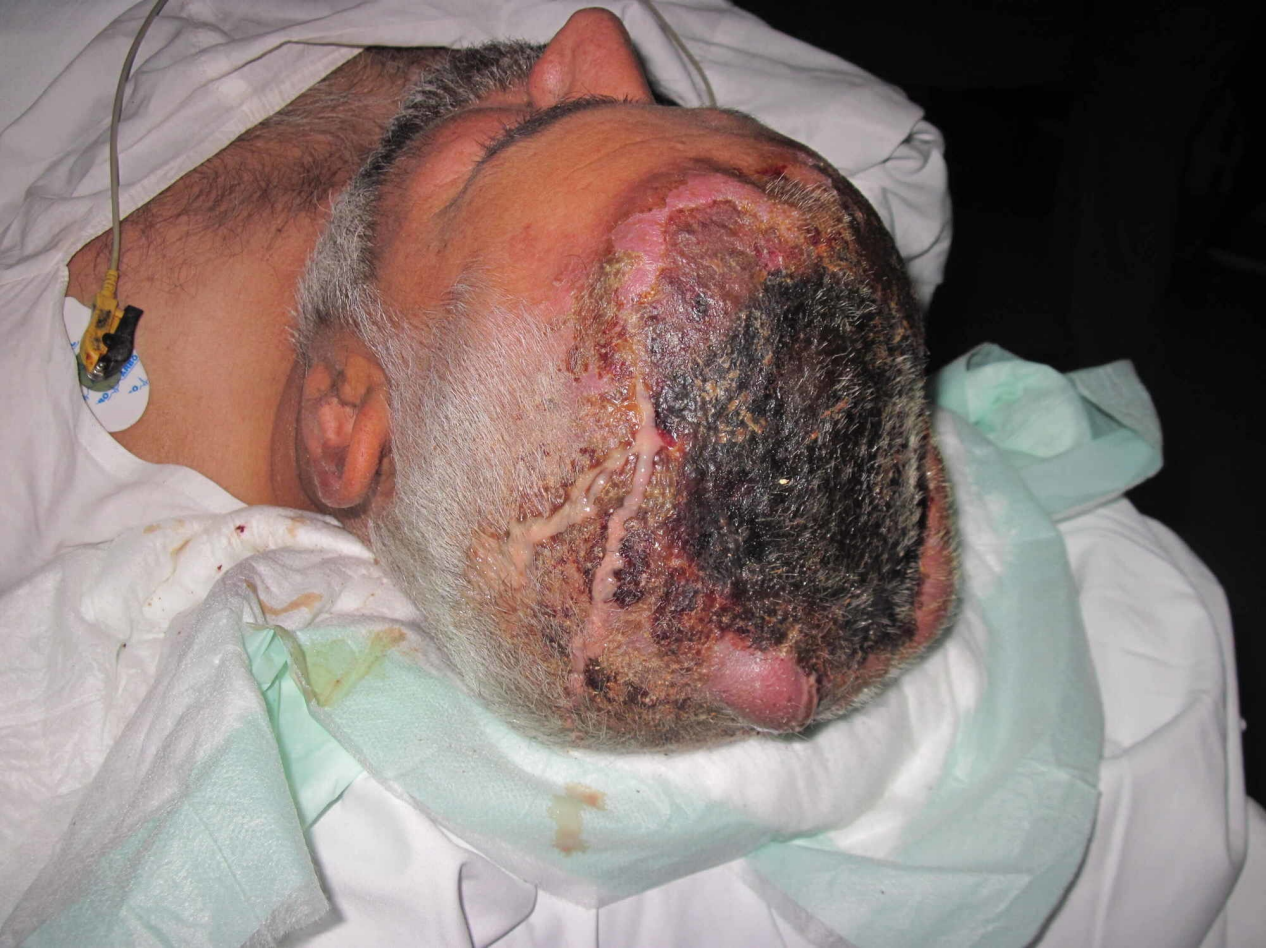
Case 4 picture showing a 40-year-old male with history of lightning strike to head presented with large necrosed area of scalp.

**Figure 15:**
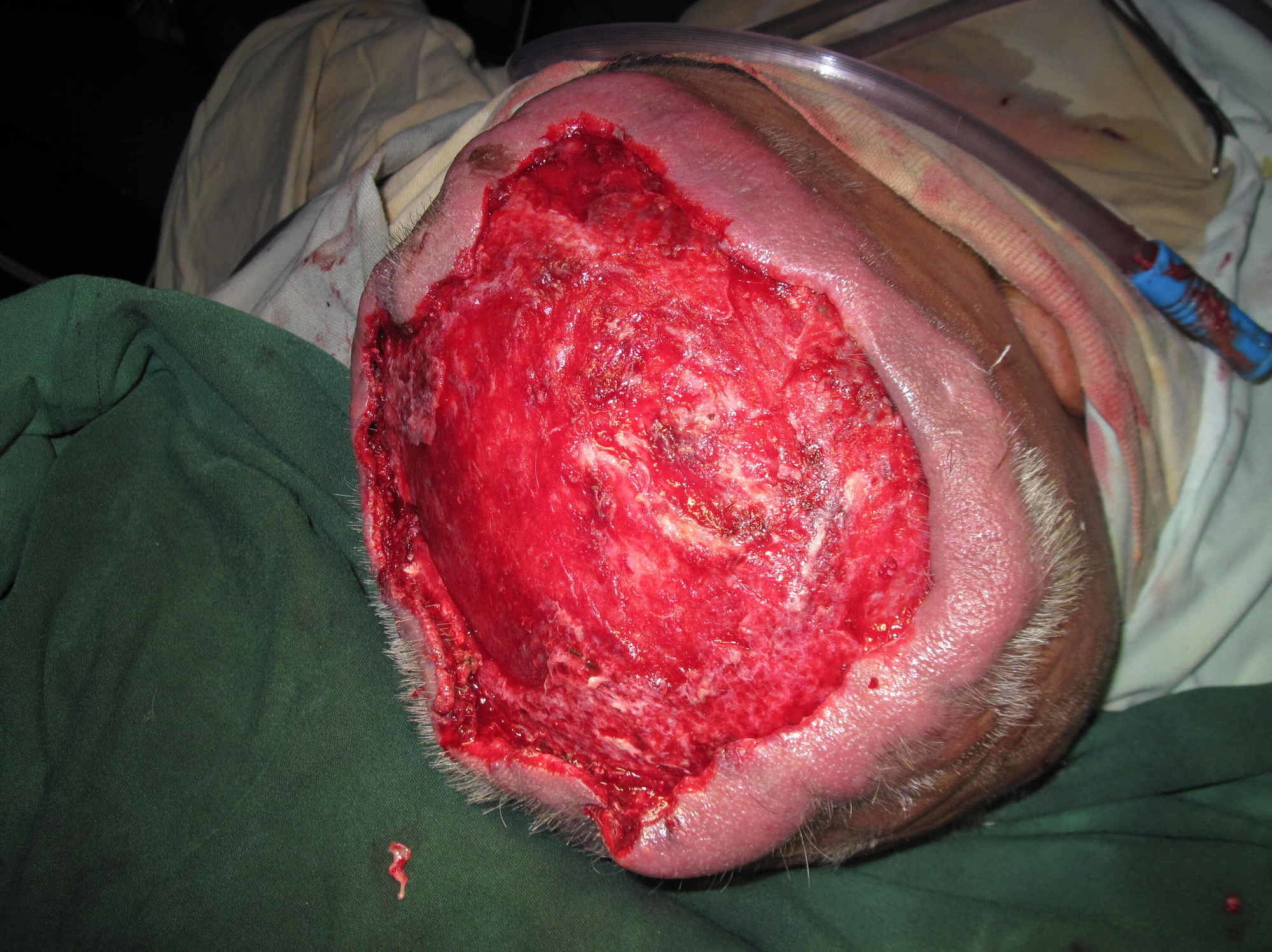
Case 4 picture showing immediately after 2nd session of debridement, wound ready for coverage.

**Figure 16:**
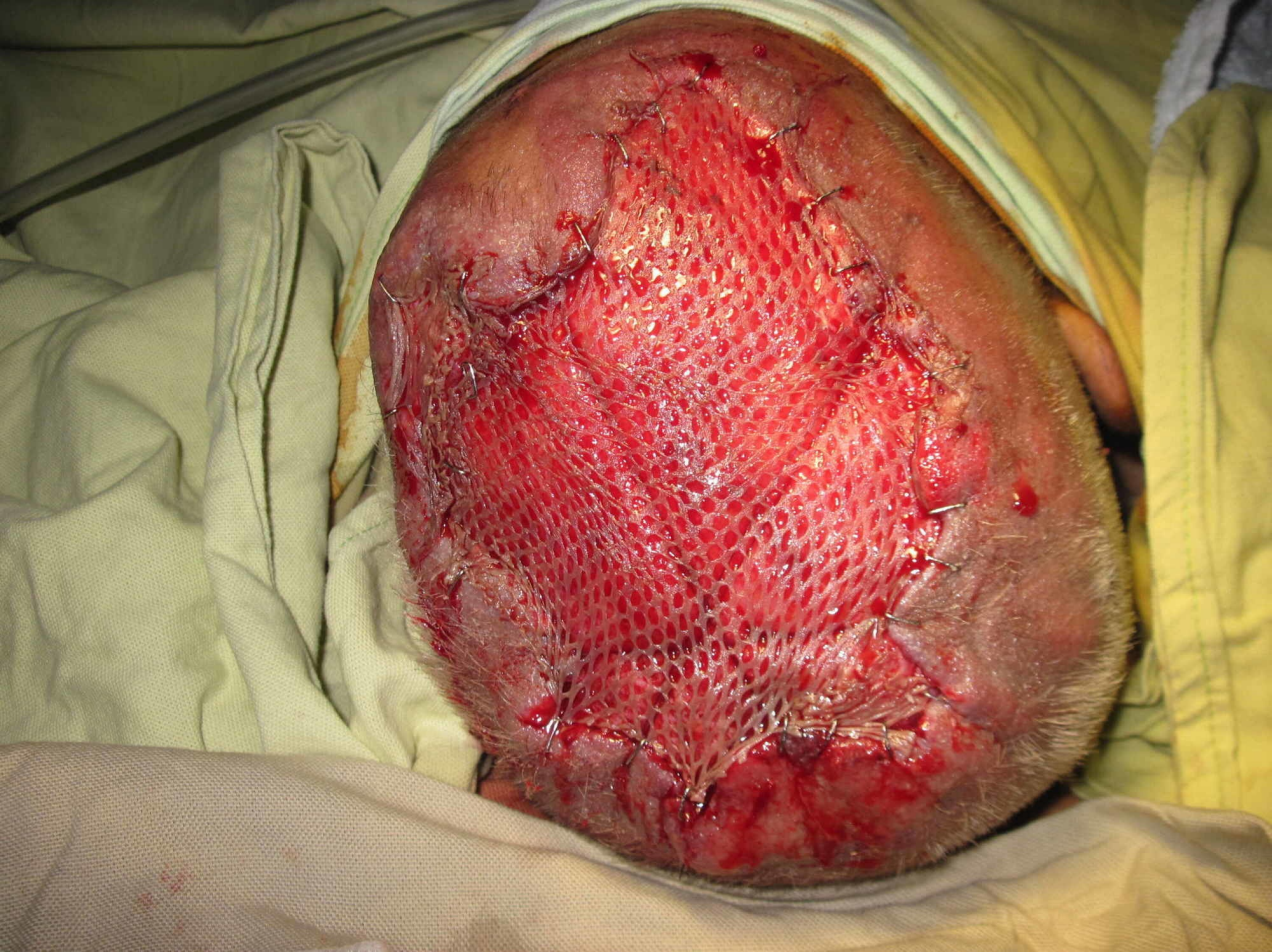
Case 4 picture showing skin grafting done which is dressed with NPWT dressing. NPWT: Negative pressure wound therapy

**Figure 17:**
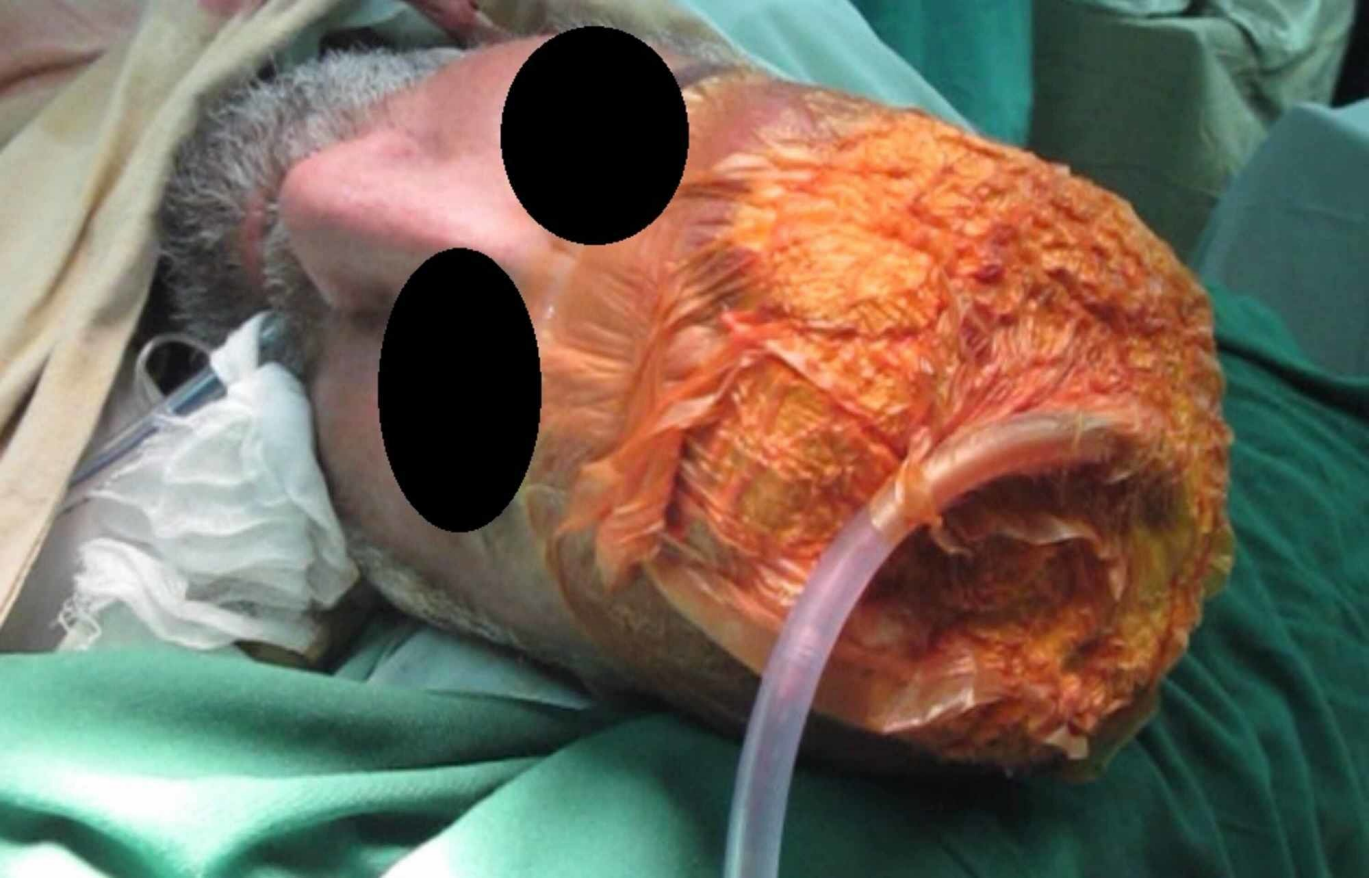
Case 4 picture showing skin grafted wound covered with NPWT dressing. NPWT: Negative pressure wound therapy

**Figure 18:**
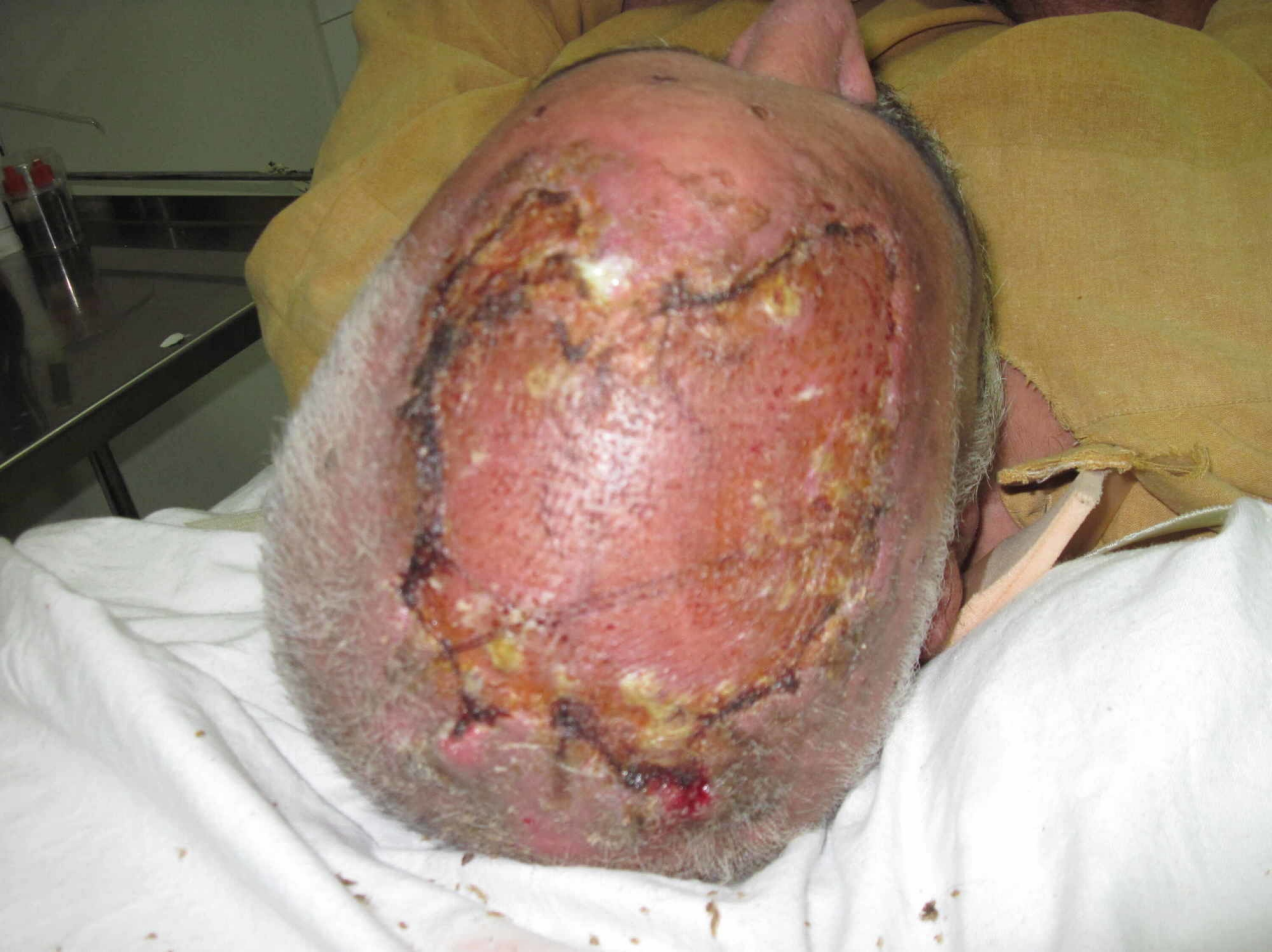
Case 4 picture showing nicely taken graft and healed wound in subsequent follow-up.

Case 4 (Figures 14-18) (Video 3) is a 40-year-old male presented with an infected wound of scalp after lightning strike. He had multiple debridements done. NPWT dressing was applied over skin grafted wound. He has nicely taken skin graft seen at follow-up pictures.

 

**Video 3:** Case 4 video showing large NPWT dressing applied to skin graft over scalp wound. NPWT: Negative pressure wound therapy

## Discussion

Management of acute wounds is a daily challenge to all the health care professionals and especially those who are working in emergency and trauma departments. All the other chronic wounds are usually transformed into acute wounds before any definitive closure or coverage is planned. KCI™(an Acelity Co., San Antonio, Texas, United States), which are the pioneers in designing the first-ever NPWT device, recommends this therapy for acute and chronic open wounds including pressure sores, grafts, flaps and dehisced wounds. This therapy is strictly contraindicated in malignancy or untreated infected wounds [8].

At first instance the general health status of the patient is optimised and the initial primary and secondary survey will lead the management plan. In all acute traumatic injuries, the goal is to reduce the bacterial load by thorough lavage of the wound and immediate debridement with bone fixation and coverage where possible. If the wound is not ready for coverage or closure it is wise to apply a negative pressure dressing which will reduce the exudate and edema of primary insult and till the patient's general health is optimised. The wound is assessed after an average of 72 hours of continuous negative pressures at our unit. Initial studies advise the use of intermittent negative pressure dressing [9]. There are studies which suggest that change of dressing may be delayed for six to seven days [10].

The basic mechanism of negative pressure dressing is mechano-transduction stimuli and deformation leading to activation of growth factors and mitosis [5,11]. This further promotes angiogenesis and new tissue formation in the form of granulation. New studies present instillation of different liquids which are antimicrobial, anti-fungal and local anaesthetic [12]. They are effective in reducing bacterial load and relieving pain.

The use of negative pressure wound therapy has been widely practised in the last two decades, in the USA and many countries [13], probably because of the increased understanding of its mechanism of action and also larger studies which support its critical role in wound management as compared to conventional dressings [14]. In the early nineties, Prof. S.A. Malik (unpublished data) introduced a local made version of negative pressure therapy in Pakistan to overcome the cost and burden of patients and hospitals. We in Pakistan receive a majority of patients who are from the low income (less than one dollar per day) category and present with a diverse variety of wounds which are either acute or chronic. The primary surgeon cannot afford any further complications and the patient cannot bear the expenses of treatment. Surgeons, constrained by these factors, have to apply this indigenous wound management substitute which gives a reliable expected outcome, thereby providing time for the patient to optimize his or her circumstances. Krticka et al. observed low complication rates and improved wound status for definitive coverage [15]. NPWT dressing if used with caution and following the guidelines provided by KCI USA, Inc., the incidence of complications (pain, fluid depletion, infection, toxic shock syndrome and bleeding) is very low [4,16-18].

Since 2010 till now we have received hundreds of patients with large open wounds, the majority of them were dealt initially with debridement and NPWT dressings. After the first or second dressings most of them were ready for coverage (graft, flap, direct closure). The average pain score was 3 out of 10 on VNS (verbal numerical scale) which is significantly low as compared to study done by Fraccalvieri et al. who compared pain associated with foam (mean pain score 6.5) and gauze (mean score 4) dressings [19].

In our patients, the complication rate was very low (less than 1%) which is comparable with the study done by Leininger [20]. The overall hospital stay and expenses were markedly reduced in the majority of our patients. This helped our patients in terms of cost and also early recovery with reduced number of dressings and procedures. Angelis et al. and Siddha et al. advocate in their study that NPWT dressings as compared to the standard dressings accelerate healing time, reduce overall cost and avoid frequent dressings which cause patients discomfort [21,22]. In the majority of our patients, the portable suction machine was provided by the plastic surgery department after auto-claving and change of filters tubing.

Apart from all these benefits we have noticed early quick graft take in uneven wound surface and improved patients’ psychological status, because the portable negative pressure machine can be easily managed at home. Stannard et al. have shown in their study, improved outcomes in grafts and skin substitutes take-rate [23]. In our patients especially those with abdominoplasty and sternal wound dehiscence, we observed reverse tissue expansion phenomenon which is a mechanical creep in-drawing force produced by negative pressure therapy. This phenomenon is previously mentioned in a study conducted by Fenn and Butler [24].

All patients after admission were thoroughly assessed, general health optimized and wounds were debrided under general anesthesia. NPWT was applied to all these patients and sent home with local made phlegm suction apparatus. In later follow-ups after 72 hours wounds were reassessed. After the assessment decision was made to close the wound or reapply NPWT dressing. Majority (>90%) of our patients’ wounds were ready for coverage after two sessions of NPWT dressings with significant reduction in C-reactive protein levels (78% cases) and micro-organisms (negative wound tissue cultures in 54% cases). Moues et al. in their study of open wounds (n = 29) showed a significant decrease (p < 0.05) in bacteria load (change of spectrum) [25]. Steingrimsson et al. observed a marked reduction in the infections after applying NPWT [26]. In another study which was done on intrathoracic applications of NPWT, the infection was not reduced in most cases [27].

NPWT is an old technique but it is time tested and it has a key role in converting a complex three-dimensional wound into a two-dimensional wound to progress towards the definitive management. In the developing countries, NPWT stays as the first line of treatment in management of complex wounds after debridement.

## Conclusions

NPWT is still proving itself in tipping points of wound management with the luxury of user-friendly properties and expected outcomes. We strongly recommend the use of NPWT (preferably original model manufactured by KCI, an Acelity Co., San Antonio, TX, USA) in complex wounds.
